# High-intensity versus low-intensity laser acupuncture in chronic, non-specific low back pain: a double-blinded, randomized controlled trial

**DOI:** 10.3389/fmed.2026.1857622

**Published:** 2026-07-15

**Authors:** Doaa Ayoub Elimy, Neama Hamed Neamat Allah, Nosiba Abdelnasser Gabarty Abdelwahed, Aliaa Mohamed Elabd, Nesma M. Allam, Engy BadrEldin S. Moustafa, Hailah M. Almohaimeed, Mustafa Shukry, Mohammed A. Gebba, Mohamed El-Sherbiny, Abdullah Obaid Alanazi, Mohamed M. Abdelfattah Abdelrahman, Aida Amir Nassif

**Affiliations:** 1 Department of Medical Rehabilitation Sciences, College of Applied Medical Sciences, Najran University, Najran, Saudi Arabia; 2 Health Research Center, Najran University, Najran, Saudi Arabia; 3 Department of Biomechanics, Faculty of Physical Therapy, Cairo University, Giza, Egypt; 4 Department of Physical Therapy for Surgery, Faculty of Physical Therapy, Cairo University, Giza, Egypt; 5 Department of Physical Therapy for Basic Sciences, Faculty of Physical Therapy, Benha University, Benha, Egypt; 6 Department of Physical Therapy and Health Rehabilitation, College of Applied Medical Sciences, Jouf University, Sakakah, Saudi Arabia; 7 Department of Physical Therapy for Neurology and Neurosurgery, Faculty of Physical Therapy, Cairo University, Cairo, Egypt; 8 College of Health Sciences, American University in the Emirates, Dubai, United Arab Emirates; 9 Department of Basic Sciences, College of Medicine, Princess Nourah bint Abdulrahman University, Riyadh, Saudi Arabia; 10 Department of Biomedical Sciences, College of Veterinary Medicine, King Faisal University, Al-Ahsa, Saudi Arabia; 11 Department of Oral Surgery and Diagnostic Sciences, Faculty of Dentistry, Applied Science Private University, Amman, Jordan; 12 Department of Basic Medical Sciences, College of Medicine, AlMaarefa University, Riyadh, Saudi Arabia; 13 The National Center for Complementary and Alternative Medicine (NCCAM), Riyadh, Saudi Arabia; 14 Department of Anesthesia, Surgical Intensive Care and Pain Management, Faculty of Medicine, Mansoura University, Mansoura, Egypt; 15 Department of Anesthesia and Critical Care, King Abdulaziz University Hospital, King Abdulaziz University, Jeddah, Saudi Arabia; 16 Department of Physical Therapy for Basic Sciences, Faculty of Physical Therapy, Cairo University, Giza, Egypt; 17 School of Health and Social Work, University of Hertfordshire, hosted by GAF (UH-GAF), Cairo, Egypt

**Keywords:** exercise therapy, laser acupuncture, low back pain, muscle fatigue, muscle strength

## Abstract

**Background:**

Chronic nonspecific low back pain (CNSLBP) is a musculoskeletal condition characterized by persistent discomfort, weak trunk muscles, exhaustion, and disability.

**Objectives:**

The purpose of this study is to compare the effects of high-intensity laser acupuncture (HILA) and low-intensity laser acupuncture (LILA), combined with exercise, on strength of trunk extensors, fatigue, level of pain, range of motion (ROM) of lumbar flexion, and disability in patients with CNSLBP.

**Materials and methods:**

60 participants with CNSLBP were divided into three groups in this randomized, double-blind, sham-controlled trial: the high-intensity laser (HILA) group, who received HILA over 15 acupuncture points along with exercises; the low-intensity laser (LILA) group, who received LILA over the same points plus exercises; and the sham group, who received sham laser in addition to exercises. The interventions were received three times/week for four consecutive weeks. The strength of trunk extensors, fatigue, pain, lumbar flexion ROM, and disability were measured at baseline, after 4 weeks, and at a 1-month follow-up. The assessment tools used included isokinetic dynamometry, the visual analogue scale (VAS), the modified Schober test (MST), and the Oswestry Disability Index (ODI).

**Results:**

Initial analysis showed that there were no statistically significant differences between the three groups (*p* > 0.05). Both the HILA and LILA groups showed substantial improvements in all outcomes posttreatment and over the follow-up period compared to the sham group (*p* < 0.05). Significantly, trunk extensor strength and pain reduction improved more in the HILA group (*p* < 0.05). However, fatigue and disability did not differ between the two active laser groups. All groups showed minor changes between the post-treatment and follow-up periods.

**Conclusion:**

Patients with CNSLBP may have a short-term benefit from HILA and LILA combined with exercise rather than from exercise alone in terms of trunk extensor strength and fatigue, pain, lumbar flexion ROM, and disability. However, both laser treatments showed comparable effects on fatigue and disability; HILA appears to be more effective than LILA in improving trunk extensor strength and relieving pain. To define the ideal treatment parameters and to evaluate long-term effects, further research is required.

**Clinical Trial Registration:**

https://clinicaltrials.gov/, identifier [NCT06280846].

## Introduction

Low back pain (LBP) is a common musculoskeletal disorder that greatly increases disability. Ninety percent of LBP cases are categorized as nonspecific, meaning that the symptoms have no discernible underlying cause. The main features of nonspecific lower back pain include pain, tightness, or stiffness in the area between the lower gluteal folds and the costal edge, which may happen with or without sciatica. Chronic nonspecific low back pain (CNSLBP) is the term used to describe symptoms that last longer than 12 weeks. Recurrence is prevalent, with approximately 69% of people having another episode within a year of recovery and 40% needing medical intervention or becoming disabled (1).

Patients with CNSLBP frequently exhibit reduced trunk muscular activation and strength in addition to persistent pain when compared to healthy people. This is associated with alterations in motor control, physical deconditioning, and arthrogenic muscle inhibition (2). These issues might worsen symptoms by decreasing muscle endurance, increasing fatigue, and limiting muscle blood flow (3). As a result, the management of LBP is a therapeutic challenge that often requires a multidisciplinary strategy (4). Restoring trunk muscle strength and function while reducing pain should be the main goals of treatment regimens (3).

The American College of Physicians (ACP) advises using non-pharmacological therapies such as exercise, acupuncture, and low-level laser therapy for the initial treatment of chronic LBP. Nevertheless, there is little evidence to promote low-level laser therapy (5). Acupuncture, which involves the stimulation of certain acupoints, has been shown to be effective in reducing pain and dysfunction related to CNSLBP (6) and improving muscle strength (7). However, using acupuncture needles may have negative consequences such as bleeding, infection, needle breakage, or needle anxiety, leading to the need for less invasive and safer alternatives (8).

Photobiomodulation (PBM), which includes both low- and high-intensity laser therapies, is considered a safe and noninvasive method for treating a variety of musculoskeletal disorders (8, 9). Low-intensity lasers, with power outputs less than 500 mW, demonstrate therapeutic photochemical reactions without producing heat because of their low irradiation levels (10). On the other hand, high-intensity lasers with outputs more than 500 mW can penetrate deeper into tissues, delivering a combination of photothermal, photochemical, and photomechanical effects. PBM is useful in the treatment of chronic musculoskeletal disorders such as spinal pain (4). Despite differences in dosage and wavelength, it can improve muscle function and reduce fatigue-related symptoms when given prior to exercise (11).

Laser acupuncture (LA) is a simple, noninvasive, and drug-free technique that uses lasers to stimulate acupoints. It serves as an alternative to conventional acupuncture, improving circulation and reducing pain and inflammation. PBM, combined with acupoint stimulation, may maximize the benefits of PBM while maintaining the acceptance of acupuncture-based treatments (8). Moreover, systematic reviews and meta-analyses have demonstrated that LA is a beneficial treatment strategy for pain management and movement rehabilitation in musculoskeletal disorders (12).

Previous research has compared high- and low-intensity laser treatments for CNSLBP. Abdelbaset et al. (13) observed no significant difference between the two laser intensities in enhancing discomfort, lumbar mobility, and impairment. However, that study examined conventional PBM administered locally across the lumbar region, not laser stimulation provided via specific acupoints. Similarly, recent reviews showed no significant differences in either laser in treating musculoskeletal problems, including LBP, while emphasizing the necessity of well-designed randomized controlled trials (RCTs) (9). Although both laser treatments can improve pain and function, evidence of LA in CNSLBP remains limited (10).

A significant gap in knowledge exists regarding the differential effects of high- and low-intensity lasers when applied via acupuncture points compared to traditional local photobiomodulation (PBM). Comparative research on high-intensity laser acupuncture (HILA) and low-intensity laser acupuncture (LILA) for chronic nonspecific low back pain (CNSLBP) is limited. Furthermore, previous laser studies have focused on subjective outcomes, such as pain and impairment, while objective assessments, particularly regarding the strength and fatigue of trunk extensor muscles, have been insufficiently explored despite their clinical relevance in low back pain (1, 3). Therefore, this study aimed to examine the effects of HILA and LILA, in conjunction with exercise, on trunk extensor strength, fatigue, pain, lumbar flexion range of motion (ROM), and disability in patients with CNSLBP.

## Materials and methods

### Study design

This randomized, double-blind, sham-controlled trial was conducted at the physical therapy outpatient clinics of Cairo University between March and October 2024. The study was approved by the Research Ethics Committee of the Faculty of Physical Therapy, Cairo University (Agreement No. P.T.REC/012/004878) and prospectively registered at ClinicalTrials.gov (NCT06280846). All procedures adhered to the standards and ethical guidelines of the 1964 Declaration of Helsinki and its subsequent amendments. Participants provided written informed consent and received a comprehensive explanation of the study procedures prior to their involvement.

### Subjects

The study involved 60 participants recruited through referrals from an independent orthopedic specialist who was not engaged in the research. The inclusion criteria were as follows: participants had to have CNSLBP persisting for over 3 months, with no pathological findings confirmed by their medical history and physical examination (14); they were required to be aged between 20 and 40 years (15); participants were required to have a body mass index (BMI) ranging from 18.5 to 24.9 kg/m^2^ to eliminate any influence of body weight; and they had to report a pain intensity of at least 30 millimeters (mm) on a standard 100 mm visual analog scale (VAS) (16).

Participants were excluded if they had undergone spinal surgery or experienced a spinal fracture within the past 6 months; had used corticosteroids, anticonvulsants, or anti-inflammatory medications; or had any neurological, infectious, rheumatologic, endocrine, psychiatric, or cognitive disorders. Additional exclusion criteria included treatment for LBP within the preceding 3 months, pregnancy, spinal stenosis, thyroid disorders, obesity, presence of a pacemaker, chronic heart disease, known photosensitivity, local injury in the treatment area, active cancer, cancer therapy within the past year, and remission from cancer.

Participants were randomly allocated into three groups, each having an equal number of individuals: the HILA group (HILA combined with exercise therapy), the LILA group (LILA combined with the exact exercise therapy), and the sham group (LA with an inactive device combined with the exact exercise therapy). Study outcomes were measured before, after the 4-weeks period of treatment, and again a month after.

### Sample size

G*Power (version 3.1.9.4) was used to calculate the sample size. An *F*-test, involving a repeated-measures ANOVA focusing on within-between interactions, was applied to determine the effect size from pilot data of the peak torque of the trunk extensors, which was 0.237. The trail required 51 participants based on a 0.05 significance level, a desired power of 90%, a correlation of 0.5 among repeated measurements, and a non-sphericity correction factor of 1. To account for an 18% expected dropout rate, the trial size was increased to 60 individuals, distributed equally into three groups.

### Randomization

Participants were allocated to three groups via computer-based randomization^1^. A permuted block design with a block size of six and an allocation ratio of 1:1:1 was used to keep equal group sizes. The randomization sequence was generated by the last author, who did not administer the treatment or outcome assessment. Allocation concealment was achieved using sealed opaque envelopes with sequential numbering, which were not opened until the baseline assessment was completed. Sixty individuals were allocated into the HILA, LILA, and sham groups (20 participants in each).

### Blinding

In this double-blind trial, both the participants and outcome evaluators were blinded to group assignments. The last author developed and maintained the treatment recognition codes while remaining unaware of therapy administration and outcome evaluation. Furthermore, the statistician was unaware of the treatment assignments until the analyses were finalized.

The participants wore protective goggles during treatment to maintain blinding and prevent them from observing the device settings or the laser. The schedule was designed to minimize interactions between individuals from different treatment groups. The HILA group was treated on different days from the LILA and sham groups. The LILA and sham groups received treatment on the same days because of the similarity in their procedures, which included identical device setup, probe application, and treatment duration. These measures were implemented to decrease the likelihood of participants distinguishing between treatments and to reduce the probability of discussing treatment details with one another.

After the last treatment session, the participants were given a questionnaire designed to check whether blinding worked. They were asked about their laser treatment (HILA, LILA, or sham); each reply was sorted into correct, incorrect, or unsure depending on the right group. The responses helped determine whether patients could distinguish between different therapies and, consequently, whether the study’s blinding techniques were successful.

### Procedures

Before participants’ enrolment, the second author conducted a screening interview and clinical assessment to ensure that they met the study’s requirements and then enrolled them in the study. During this first meeting, we explained the study, what would happen, and any possible risks. Participants provided written consent after receiving all the details. Eligible patients then set up times for their baseline assessment, treatment sessions, post-treatment evaluation, and follow-up visits.

### Intervention

After the baseline evaluation, the final author opened the envelopes and allocated the participants to their respective intervention groups. Before administering the treatment, a familiarization session was held to instruct the participants on the exercises and their proper execution, ensuring consistency. Each participant then received their assigned intervention, which involved either actual laser therapy (HILA or LILA) or sham treatment with identical supervised exercises.

### Laser acupuncture

During the study, the room temperature was consistently maintained at 20 °C. To ensure safety and minimize bias during treatment, both the researcher (identified as the third author) and the participants wore protective goggles. The participants were positioned face down, with padding placed under their heads and heels to ensure comfort. Prior to treatment application, the researcher, who possessed over a decade of experience and was aware of each participant’s group assignment, cleansed the treatment area using an alcohol pad.

**FIGURE 1:**
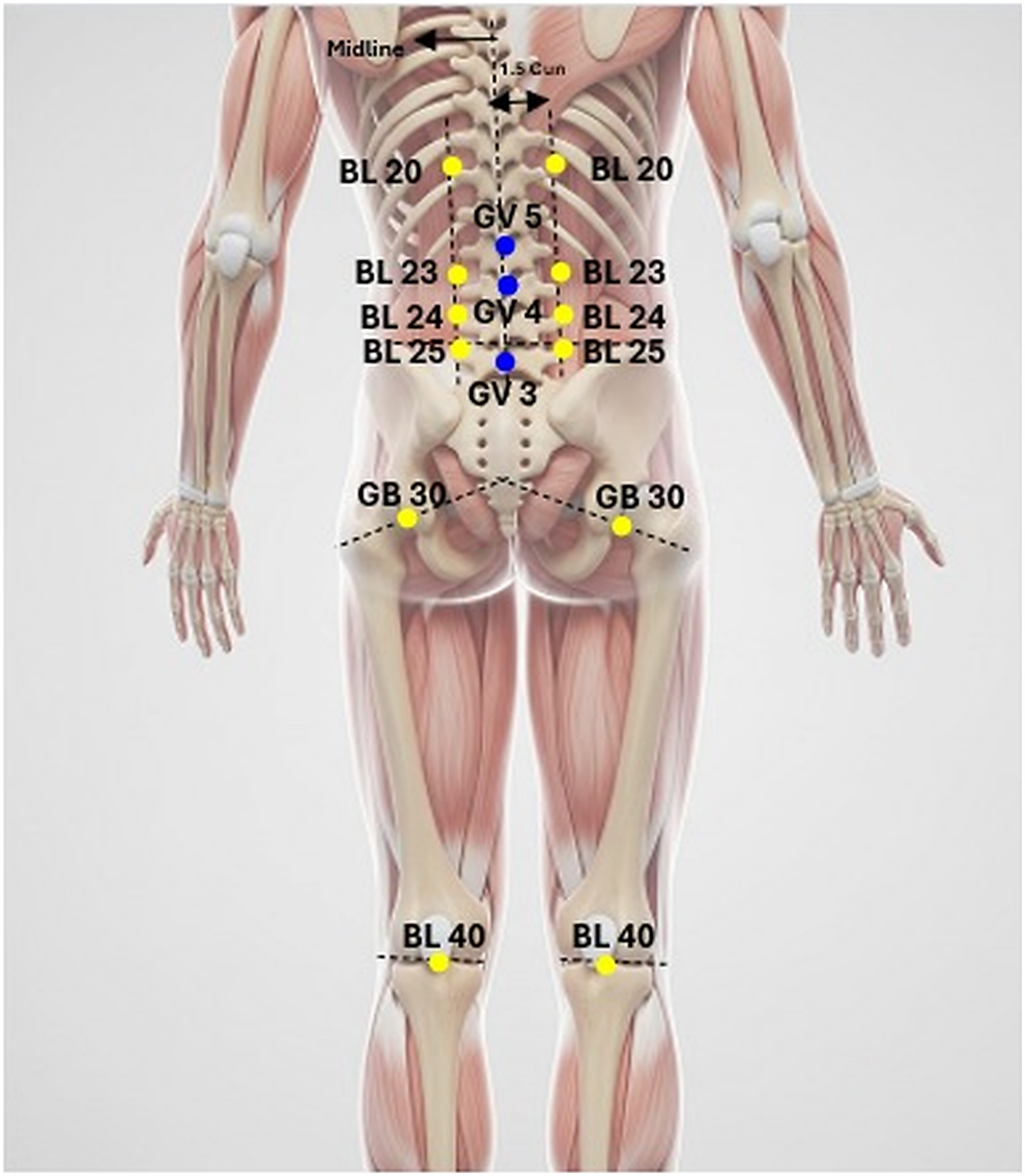
Acupoints used for laser acupuncture treatment.

Laser treatment was conducted perpendicularly to the 15 predefined acupoints, including bilateral points (BL20, BL23, BL24, BL25, BL40, and GB30) and three midline points (GV3, GV4, and GV5), based on prior research (16, 17). The placement of these points was defined according to the World Health Organization guidelines (18) (Table 1). To ensure consistent administration in treatment and facilitate comparison among the HILA, LILA, and sham groups, the same 15 points were used for all participants in the following sequence: unilateral GV3, GV4, and GV5, followed by bilateral BL20, BL23, BL24, BL25, BL40, and GB30 (Figure 1). Consequently, the intervention involved laser stimulation of predefined acupuncture/meridian points aligned with Traditional Chinese Medicine locations, rather than individualized traditional acupuncture needling.

**TABLE 1:** Anatomical locations of the acupuncture points used in the current study.

Acupuncture point	Location description
BL20	Located at the level of the 11th thoracic vertebra (T11), 1.5 B-cun lateral to the posterior median line in the upper back region.
BL23	Located at the level of the 2nd lumbar vertebra (L2), 1.5 B-cun lateral to the posterior median line in the lumbar region.
BL24	Positioned at the level of the 3rd lumbar vertebra (L3), 1.5 B-cun lateral to the posterior median line in the lumbar region.
BL25	Located at the level of the 4th lumbar vertebra (L4), 1.5 B-cun lateral to the posterior median line in the lumbar region.
BL40	It is situated at the midpoint of the popliteal crease (back of the knee).
GB30	Located in the gluteal region at the junction of the lateral one-third and medial two-thirds of the line connecting the greater trochanter with the sacral hiatus.
GV3	In the lumbar region, just below the spinous process of the fourth lumbar vertebra (L4) along the posterior median line.
GV4	In the lumbar region, just below the spinous process of the second lumbar vertebra (L2), along the posterior median line.
GV5	In the lumbar region, just below the spinous process of the first lumbar vertebra (L1), along the posterior median line.

The comparison between HILA and LILA was based on mechanisms rather than on dose equivalence. Thus, the laser protocols were designed to vary in terms of power output, exposure duration, and total energy delivered. HILA was selected to provide higher energy applied in a shorter time, whereas LILA offers lower-energy, non-thermal, biologically modulatory stimulation over a longer period. This approach highlights the clinical distinction between high- and low-intensity laser applications rather than attempting to equalize the total energy output between the groups.

In the HILA group, the laser device produced by Zimmer MedizinSysteme, Germany (serial numbers 15200013306 and 4682), was used. This laser of infrared light with a wavelength ranging from 810 to 980 nm uses a probe of 5 mm in diameter and a spot size of 0.2 cm^2^, which could be expanded to 3.1 cm^2^ with the application of a spacer. The operational parameters were a 50% duty cycle, 5 watts of power, and 5 Hz frequency. For each spot, 60 joules (J) were delivered in a duration of 13 s, giving a fluence of about 19.35 J/cm^2^ with a spacer of 3.1 cm^2^. In each treatment session, the whole acupoint delivered a cumulative energy of 900 J, with a total treatment duration of 195 s.

In the LILA group, the ASTAR PhysioGo 701I device (serial no. PHG701I-04/S3/AX, ASTAR, Poland) was used. This apparatus emitted an infrared laser at a wavelength of 808 nm with an output power of 200 mW in continuous mode. The probe was applied directly to the skin, delivering an energy of 7.4 J at each point within a duration of 37 s, for a fluence of 15 J/cm^2^. The whole energy delivered across the same 15 acupuncture points was approximately 111 J per session, with an overall treatment duration of 555 s.

The rationale for the penetration depth was based on the differences in powers and wavelength of the two laser types. A high-intensity laser, characterized by its high power, was chosen for its capacity to provide better stimulation and deeper tissue penetration (9). In contrast, low-intensity lasers, of low power and no thermal sensations, operate with low irradiation (10). Although the penetration depth was not directly measured *in vivo*, the selection of wavelengths, power, and fluences was guided by the predicted tissue interactions and clinical applications of each laser modality.

To ensure standardization of treatment, identical predetermined points of acupuncture and their therapy sequence, individual position, skin preparation, perpendicular probe placement, exercise regimen, and a 4-weeks treatment duration were applied across all groups. The same researcher performed all laser treatment sessions, and one physiotherapist delivered the supervised exercise program to minimize variability in treatment.

To maintain participant blinding regarding their therapies, all auditory signals from the device, such as those for initiation, ending, or warnings, were disabled in each session. The sham treatment was applied under the exact schedule and conditions like the LILA group, utilizing the same device, points, probe placement, and session duration. The only variation was that the laser was deactivated, causing no energy delivery, and the device sounds were muted throughout all sessions. All therapies were conducted thrice weekly for 4 weeks.

### The exercise program

All participants followed the identical exercise program throughout the study. The same physiotherapist supervised the program without being aware of the group assignments or whether the participants received laser therapy to reduce potential bias. The participants exercised thrice a week for 4 weeks.

The exercise plan included stretching for the calves, hamstrings, and back (19). Each stretch was sustained for 30 s and repeated four times (20). The lumbar stability training aimed to fortify the deep muscles. Exercises to strengthen the back and abdominal muscles of the participants were also performed. Each exercise was done for 10 s, then a 10-s rest, and repeated ten times. The workouts were adapted to the participants’ capabilities (21) to keep safety and enhance muscular strength.

### Outcome measures

The initial baseline data included participants’ clinical and demographic information, such as age, height, weight, BMI, symptom duration, marital and employment status, pain levels, peak torque, fatigue, lumbar flexion ROM, and disability. The primary outcome measure was trunk extensor strength, which was assessed using an isokinetic dynamometer. Secondary outcomes included trunk extensor fatigue, evaluated using an isokinetic dynamometer; pain, assessed using the VAS; lumbar flexion ROM, measured using the modified Schober test (MST); and disability, evaluated using the Oswestry Disability Index (ODI). All outcomes were assessed at baseline, following the 4-weeks program, and 1 month later.

### Primary outcome measure

#### Trunk extensor strength

Trunk extensor strength was assessed using a Biodex isokinetic dynamometer (Biodex Medical Systems, Shirley, NY, USA) that was calibrated prior to the experiment. Initially, the participants were familiarized with the test procedure (22). Participants were then seated on the dual-position back extension/flexion attachment with their trunks erect, hips and knees flexed at 90°, and their thighs parallel to the ground. The axis of rotation of the dynamometer was aligned with the imaginary line between the anterior superior iliac spines (ASIS), serving as the anatomical reference position. To secure the participant to the back extension/flexion attachment, padded surfaces were positioned around the head, sacrum, upper trunk, and anterior aspect of the tibia, and Velcro straps were applied to the upper trunk, thighs, and pelvis. Trunk movement during testing was restricted to a range of 30° (−30°) of flexion to 20° (+20°) of extension from the anatomical reference position (0°), resulting in a total trunk movement range of 50°. This limited trunk motion ensured the isolation of lumbar motion and minimized hip flexion and extension involvement. Furthermore, the placement of the axis of rotation of the dynamometer at the ASIS level, along with the posterior sacrum pad and pelvic strap, reduced hip joint movement during the procedure. Prior to testing, the participants completed one set of 10 submaximal trunk flexion-extension repetitions at the test angular velocity as a warm-up, facilitating preparation for the test procedure (23). The participants then performed maximal trunk extension efforts at an angular velocity of 60°/s with their hands positioned across their chest. The data analyzed included peak torque results from these maximal efforts. All participants completed the tests without complaints (22).

### Secondary outcome measures

#### Fatigue of trunk extensors

The protocol for a 50% torque reduction was applied to assess trunk extensor fatigue, identifying the point of significant muscular fatigue based on each participant’s peak torque (24, 25). Participants were seated in a standardized position on the back extension/flexion attachment, maintaining an upright position, with their knees and hips flexed at 90°, thighs horizontal, and the axis of the dynamometer aligned with the ASIS level. The trunk, pelvis, thighs, and legs were immobilized by using pads and Velcro straps to ensure the same restricted trunk range in the strength test (23). The participants were told to do maximal isokinetic concentric trunk extensions and submaximal flexions at a speed of 60°/s (25). Peak torque was recorded throughout the extension with verbal encouragement to maximize the effort. The test was continued until the torque dropped below 50% of the initial peak level for three consecutive attempts. The total number of attempts required to reach the “50% peak torque” was counted as a value of fatigue (24).

### Pain assessment

The VAS was employed for determining pain severity. This scale is made up of a 100 mm-long horizontal line with “no pain” on one end and “severe pain” on the other. The participants were required to make marks on this line that accurately represented their highest level of pain. The pain score was calculated by measuring the distance in mm between the no-pain end and the participant’s marks (26).

### Lumbar flexion ROM

The MST was used by first identifying the lumbosacral junction through the dimples of Venus’s location. A horizontal line was drawn connecting the top edges of the dimples to serve as a reference point. Two additional lines were marked: one approximately 10 cm above and the other approximately 5 cm below the reference. The range of lumbar flexion was estimated as the variations between the readings taken when standing upright and bending forward (27).

### Disability

The Arabic ODI was utilized to assess the disability of patients with LBP. It comprises ten sections: one section measures pain intensity, while the other nine assess daily living activities, including lifting, walking, socializing, performing personal care tasks, sitting, standing, sleeping, traveling, and sexual activity. Each item is scored on a scale of 0 (indicating no disability) to 5 (indicating maximum disability). The total score was derived by summing the scores of all ten sections, dividing by 50 (the highest possible score), and multiplying by 100. A higher index score indicates a greater degree of disability (28).

### Adverse events and safety monitoring

During the study, the participants were continuously monitored for potential adverse effects on their health and safety throughout the therapy and follow-up periods. They were instructed to report any unusual or abnormal signs or symptoms to the study researcher. Both high- and low-level laser therapies were evaluated for potential side effects using participant self-reports, clinical assessments conducted by the research staff, and participant medical histories.

After each treatment session, the research staff asked the patients whether they had experienced any adverse effects due to treatment (increased pain, redness, burning of skin, swelling, dizziness, headache, fatigue, or any other adverse effect to the local or systemic system). The reported events were documented (type, time of occurrence, duration of occurrence, and severity) and reviewed by the study investigator. The research team also performed clinical observations of all participants before and after each treatment procedure, looking for signs of adverse effects.

If a participant experienced a severe adverse reaction or side effects, treatment was temporarily stopped until further clinical evaluation. Participants who exhibited significant side effects from the treatment intervention were withdrawn from the research.

### Statistical analysis

Data analysis was done by using SPSS software version 25 (IBM, Chicago, Illinois, USA). Categorical variables were presented as frequencies and percentages [N (%)], and group comparisons were performed using the Chi-square test (χ^2^). For continuous data, the Shapiro–Wilk test was applied to test data normality, and data were expressed as mean ± standard deviation (SD). Homogeneity of data was tested using Levene’s test, while one-way ANOVA was applied for comparing more than two groups. A mixed repeated-measures ANOVA was used to evaluate the effects of a between-subjects factor (treatment) and a within-subjects factor (time) on a continuous dependent variable, incorporating Bonferroni adjustments for *post hoc* analyses of multiple comparisons. Mean differences among all groups, together with 95% confidence intervals, were estimated using the *t*-test. Effect sizes were measured by partial eta squared (ηp^2^) values. Statistical significance was set at *p* < 0.05.

## Results

### Demographic and subject characteristics

**FIGURE 2:**
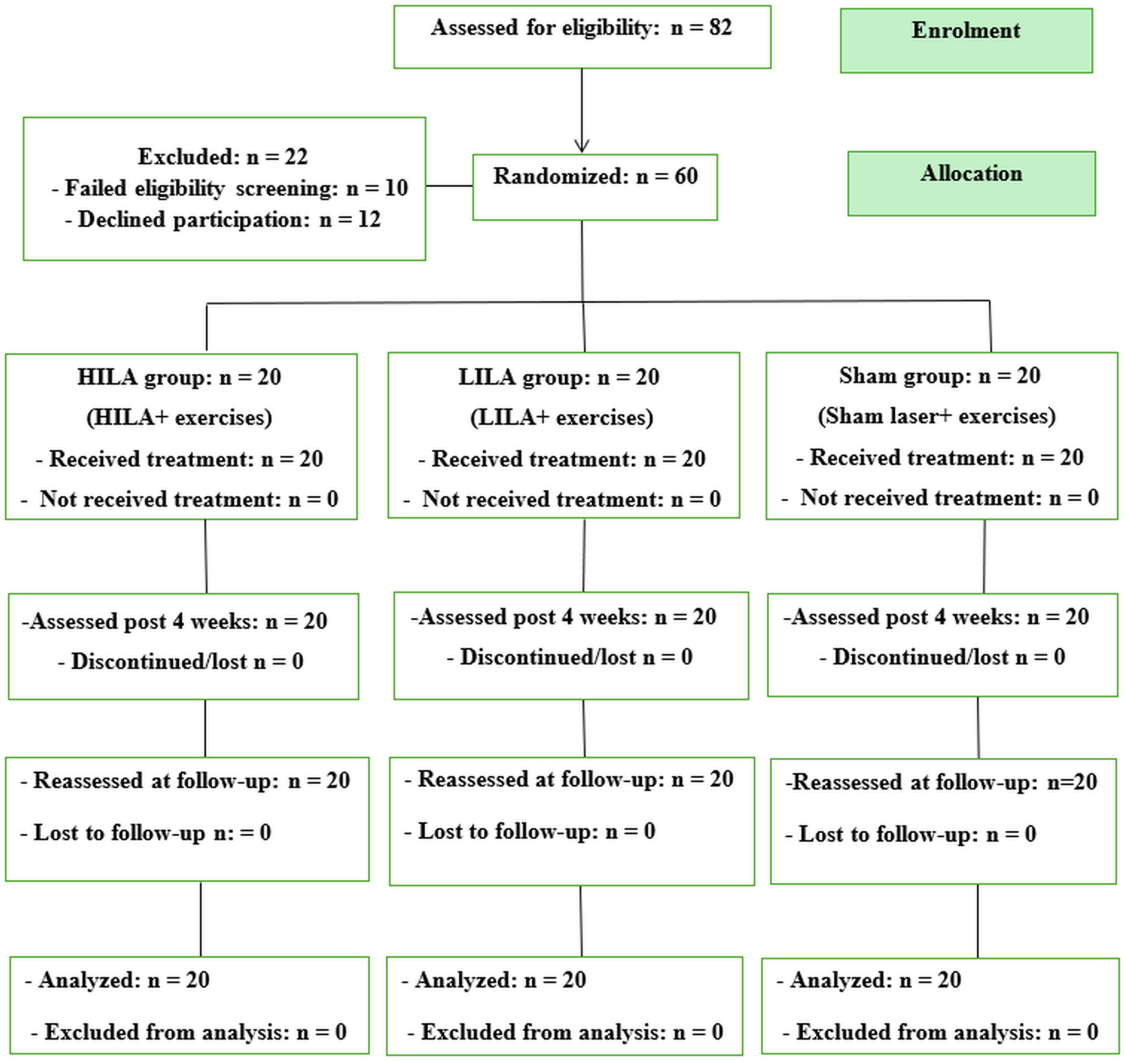
Participant flow diagram according to CONSORT recommendations.

Among the 82 participants assessed for eligibility, 22 were excluded because they did not meet the inclusion criteria (10 participants) or declined to participate (12 participants). Consequently, 60 participants were randomly assigned to three groups: HILA, LILA, and Sham, with 20 individuals in each group (Figure 2). The final analysis included only participants who adhered to their treatment plans. The baseline characteristics of the three groups are presented in Table 2, and no significant differences were observed among them (*P* > 0.05).

**TABLE 2:** Baseline characteristics of the three groups.

Variables	HILA group	LILA group	Sham group	Test of significance	*P*-value
Age (years)	32.25 ± 4.61	31.90 ± 5.38	32.05 ± 4.91	*F* = 0.025	0.975
Sex, N (%)					
Male	7 (35.0%)	8 (40.0%)	9 (45.0%)	χ^2^ = 0.417	0.812
Female	13 (65.0%)	12 (60.0%)	11 (55.0%)		
Weight (Kg)	69.31 ± 2.97	70.52 ± 3.43	70.79 ± 2.97	*F* = 1.257	0.292
Height (cm)	174.68 ± 2.55	173.65 ± 2.82	174.34 ± 2.90	*F* = 0.725	0.489
BMI (Kg/m^2^)	22.69 ± 1.31	23.31 ± 1.35	23.26 ± 1.19	*F* = 1.427	0.248
Duration of LBP (months)	15.88 ± 3.96	15.48 ± 4.28	15.20 ± 4.06	*F* = 0.139	0.870
Marital status, N (%)					
Single	9 (45.0%)	8 (40.0%)	9 (45.0%)	χ^2^ = 0.136	0.934
Married	11 (55.0%)	12 (60.0%)	11 (55.0%)		
Employment status, N (%)					
Employment	17 (85.0%)	16 (80.0%)	16 (80.0%)	χ^2^ = 0.223	0.895
Unemployment	3 (15.0%)	4 (20.0%)	4 (20.0%)		
Pain (mm)	69.45 ± 9.58	70.80 ± 10.66	68.05 ± 8.44	*F* = 0.410	0.666
Peak torque (N.m)	117.76 ± 9.14	119.71 ± 11.88	118.96 ± 10.93	*F* = 0.169	0.845
Fatigue (Reps)	26.90 ± 2.45	28.40 ± 5.45	27.50 ± 4.06	*F* = 0.655	0.523
Lumbar flexion ROM (cm)	4.03 ± 0.54	4.31 ± 0.77	4.26 ± 0.66	*F* = 1.036	0.361
Disability (%)	34.35 ± 4.49	32.55 ± 5.42	33.35 ± 5.54	*F* = 0.608	0.548

Data are presented as mean ± standard deviation and *N* (%) for categorical data. F, one-way ANOVA test; χ^2^, Chi-square analysis; *P* < 0.05, statistically significant; BMI, body mass index; LBP, low back pain; ROM, range of motion.

### Effect of treatment on peak torque, fatigue, pain, lumbar flexion ROM, and disability

For peak torque, there were statistically significant main effects for time (*F* = 718.002, *p* < 0.001, ηp^2^ = 0.926), treatment (*F* = 12.658, *p* < 0.001, ηp^2^ = 0.308), and interaction between time and treatment (*F* = 66.216, *p* < 0.001, ηp^2^ = 0.699). Within-group comparison revealed significant increases from pre- to post-in all tested groups (*p* < 0.001), favoring the HILA group over the LILA (*p* = 0.001) and the sham group (*p* < 0.001). At follow-up, peak torque significantly declined in all groups (*p* < 0.001), with the HILA group showing more improvement than the LILA (*p* = 0.001) and sham groups (*p* < 0.001) (Table 3 and Supplementary Table 1).

**TABLE 3:** Comparison of peak torque and fatigue among the three groups in the pre-, post-, and follow-up periods.

Variables	HILA group	LILA group	Sham group	*P*-value^b^	MD (95% CI)	*Post hoc*	Time × group interaction	
							*P*-value	η p^2^
Peak torque (N.m)								
Pre	117.76 ± 9.14	119.71 ± 11.88	118.96 ± 10.93	0.845	−1.96 (−10.10, 6.19)	–	<0.001*	0.699
					−1.20 (−9.35, 6.95)	–		
					0.76 (−7.39, 8.90)	–		
Post	154.71 ± 6.97	143.07 ± 10.61	131.71 ± 10.47	<0.001*	11.65 (4.42, 18.87)	P1 = 0.001*		
					23.00 (15.77, 30.23)	P2 < 0.001*		
					11.36 (4.13, 18.58)	P3 = 0.001*		
Follow-up	153.01 ± 6.00	141.93 ± 10.77	130.16 ± 10.59	<0.001*	11.08 (3.94, 18.22)	P1 = 0.001*		
					22.85 (15.71, 29.99)	P2 < 0.001*		
					11.77 (4.63, 18.91)	P3 = 0.001*		
*P*-value^a^	<0.001*	<0.001*	<0.001*					
Fatigue (Reps)								
Pre	26.90 ± 2.45	28.40 ± 5.45	27.50 ± 4.06	0.523	−1.50 (−4.67, 1.67)	–	<0.001*	0.301
					−0.60 (−3.77, 2.57)	–		
					0.90 (−2.27, 4.07)	–		
Post	45.60 ± 5.59	43.80 ± 5.66	36.70 ± 5.46	<0.001*	1.80 (−2.44, 6.04)	P1 = 0.567		
					8.90 (4.66, 13.14)	P2 < 0.001*		
					7.10 (2.86, 11.34)	P3 < 0.001*		
Follow-up	43.60 ± 5.74	41.90 ± 6.22	34.85 ± 5.16	<0.001*	1.70 (−2.66, 6.06)	P1 = 0.618		
					8.75 (4.39, 13.11)	P2 < 0.001*		
					7.05 (2.69, 11.41)	P3 = 0.001*		
*P*-value^a^	<0.001*	<0.001*	<0.001*					

Data are presented as mean ± SD. MD, mean difference; CI, confidence interval; ηp^2^, partial eta squared; P1, HILA group vs. LILA group; P2, HILA group vs. Sham group; P3, LILA group vs. Sham group;

*P*-value^a^: within-group comparison;

*P*-value^b^: between-group comparison;

*Statistically significant at *p*-value < 0.05.

For fatigue analysis, statistically significant main effects were identified for time (*F* = 255.417, *p* < 0.001, ηp^2^ = 0.818), treatment (*F* = 10.264, *p* < 0.001, ηp^2^ = 0.265), and interaction between time and treatment (*F* = 12.271, *p* < 0.001, ηp^2^ = 0.301). All three groups showed significant improvement from pre- to post-intervention, followed by a significant decline at the follow-up (*p* < 0.001). The HILA and LILA groups demonstrated significantly greater improvements than the sham group at both post-intervention (*P* < 0.001) and follow-up (*P* < 0.001 and *P* = 0.001). No significant differences were observed between the HILA and LILA groups at either post-intervention (*P* = 0.567) or follow-up (*P* = 0.618) (Table 3 and Supplementary Table 1).

Table 4 shows the statistically significant main effects of time (*F* = 360.050, *p* < 0.001, ηp^2^ = 0.863) and treatment (*F* = 40.438, *p* < 0.001, ηp^2^ = 0.587) on pain, as well as a significant interaction effect between time and treatment (*F* = 40.681, *p* < 0.001, ηp^2^ = 0.588). Postintervention pain was significantly reduced in all three groups compared to baseline (*p* < 0.001), with the HILA group showing greater improvement than the LILA (*p* = 0.007) and sham groups (*p* < 0.001). Nevertheless, significant increases in pain were noted at follow-up, although the HILA group continued to exhibit significantly lower scores than the LILA (*p* = 0.013) and sham groups (*p* < 0.001) (Supplementary Table 1).

**TABLE 4:** Comparison of pain, lumbar flexion ROM, and disability among the three groups pre-, post-, and follow-up.

Variables	HILA group	LILA group	Sham group	*P*-value^b^	MD (95% CI)	*Post hoc*	Time × group interaction	
							*P*-value	η p^2^
Pain (mm)								
Pre	69.45 ± 9.58	70.80 ± 10.66	68.05 ± 8.44	0.666	−1.35 (−8.66, 5.96)|	–	<0.001*	0.588
					1.40 (−5.91, 8.71)	–		
					2.75 (−4.56, 10.06)	–		
Post	28.30 ± 5.37	35.25 ± 8.13	54.90 ± 6.94	<0.001*	−6.95 (−12.21, −1.69)	P1 = 0.007*		
					−26.60 (−31.86, −21.34)	P2 < 0.001*		
					−19.65 (−24.91, −14.39)	P3 < 0.001*		
Follow-up	34.70 ± 5.57	40.95 ± 8.14	59.85 ± 6.12	<0.001*	−6.25 (−11.35, −1.15)	P1 = 0.013*		
					−25.15 (−30.25, −20.05)	P2 < 0.001*		
					−18.90 (−24.00, −13.80)	P3 < 0.001*		
*P*-value^a^	<0.001*	<0.001*	<0.001*					
Lumbar flexion ROM (cm)								
Pre	4.03 ± 0.54	4.31 ± 0.77	4.26 ± 0.66	0.361	−0.28 (−0.78, 0.22)	–	<0.001*	0.460
					−0.23 (−0.74, 0.27)	–		
					0.04 (−0.46, 0.55)	–		
Post	6.42 ± 0.43	6.00 ± 0.38	5.38 ± 0.44	<0.001*	0.42 (0.10, 0.74)	P1 = 0.007*		
					1.04 (0.72, 1.36)	P2 < 0.001*		
					0.62 (0.30, 0.94)	P3 < 0.001*		
Follow-up	6.13 ± 0.40	5.84 ± 0.38	5.12 ± 0.47	<0.001*	0.29 (−0.03, 0.61)	P1 = 0.080		
					1.01 (0.69, 1.32)	P2 < 0.001*		
					0.72 (0.40, 1.03)	P3 < 0.001*		
*P*-value^a^	<0.001*	<0.001*	<0.001*					
Disability (%)								
Pre	34.35 ± 4.49	32.55 ± 5.42	33.35 ± 5.54	0.548	1.80 (−2.13, 5.73)	–	<0.001*	0.468
					1.00 (−2.93, 4.93)	–		
					−0.80 (−4.73, 3.13)	–		
Post	19.65 ± 4.43	22.05 ± 3.05	28.30 ± 4.49	<0.001*	−2.40 (−5.48, 0.68)	P1 = 0.155		
					−8.65 (−11.73, −5.57)	P2 < 0.001*		
					−6.25 (−9.33, −3.17)	P3 < 0.001*		
Follow-up	20.90 ± 4.62	24.00 ± 2.87	30.05 ± 4.84	<0.001*	−3.10 (−6.30, 0.10)	P1 = 0.059		
					−9.15 (−12.35, −5.95)	P2 < 0.001*		
					−6.05 (−9.25, −2.85)	P3 < 0.001*		
*P*-value^a^	<0.001*	<0.001*	<0.001*					

Data are presented as mean ± SD. MD, mean difference; CI, confidence interval; ηp^2^, partial eta squared; P1, HILA group vs. LILA group; P2, HILA group vs. Sham group; P3, LILA group vs. Sham group;

*P*-value^a^: within-group comparison;

*P*-value^b^: between-group comparison;

*Statistically significant at *p*-value < 0.05; ROM, range of motion.

Regarding the lumbar flexion ROM, statistically significant main effects were found for time (*F* = 423.953, *p* < 0.001, ηp^2^ = 0.881) and group (*F* = 10.699, *p* < 0.001, ηp^2^ = 0.273), along with a time-treatment interaction (*F* = 24.304, *p* < 0.001, ηp^2^ = 0.460). A statistically significant increase was observed in all three groups post-intervention compared to pre-intervention, favoring the HILA group (*p* < 0.001) compared to the LILA (*p* = 0.007) and sham groups (*p* < 0.001). In contrast, a statistically significant decline was observed at follow-up compared to post-intervention (*p* < 0.001). The HILA and LILA groups maintained significantly higher levels than the sham group at follow-up (*p* < 0.001), with no significant difference between the HILA and LILA groups (*p* = 0.080) (Table 4 and Supplementary Table 1).

There were statistically significant main effects of disability on time (*F* = 207.875, *p* < 0.001, ηp^2^ = 0.785), group (*F* = 10.960, *p* < 0.001, ηp^2^ = 0.278), and interaction between time and treatment (*F* = 25.103, *p* < 0.001, ηp^2^ = 0.468). Disability scores showed significant decreases in all three groups post-intervention (*p* < 0.001), with the HILA and LILA groups showing significantly greater improvements than the sham group (*p* < 0.001). At follow-up, all groups showed a significant increase (*p* < 0.001), while the HILA and LILA groups had lower scores than the sham group (*p* < 0.001) (Table 4). However, the HILA and LILA groups did not differ in terms of post-intervention (*p* = 0.155) or follow-up (*p* = 0.059) (Supplementary Table 1).

### Blinding effectiveness

The blinding of participants was measured using Bang’s Blinding Index (BI). The HILA group exhibited a BI of −0.05, derived from nine correct, ten incorrect, and one unsure response among its 20 participants. The LILA group demonstrated a BI of −0.15, based on seven correct, ten incorrect, and three unsure responses from its 20 participants. The sham group recorded a BI of −0.20, with five correct, nine incorrect, and six unsure responses among its 20 participants. In all three groups, the number of incorrect guesses was higher than the number of correct guesses, demonstrating that blinding was appropriately maintained, as seen by the negative BI values being close to zero.

### Adverse effects

No significant or lasting side effects were observed during the period of treatment or follow-up, and no participant dropped out from the study. The side effects were mild and resolved quickly. After the first laser treatment session, mild redness at the site of the laser was observed among five patients who received HILA treatment and two who received LILA treatment; however, it resolved spontaneously without any medical intervention. No reports indicated excessive swelling, dizziness, headaches, fatigue, increased pain, or other systemic side effects, suggesting that both LA treatments were well tolerated by the participants.

## Discussion

The current findings indicated that both the HILA and LILA groups were considerably better than the sham group (*p* < 0.05) across all measured outcomes. In comparing the two active laser treatments, HILA resulted in superior improvements in trunk extensor strength and pain relief both post-treatment and follow-up time points (*p* < 0.05). Regarding lumbar flexion ROM, HILA demonstrated greater enhancement immediately post-treatment; however, this difference was not sustained at the period of follow-up. No significant variation was observed among the HILA and LILA groups in trunk extensor fatigue or disability at any time point. Furthermore, all groups exhibited minor changes in their outcomes from post-treatment to follow-up, across all measures.

The increase in trunk extensor strength observed in both the HILA and LILA groups compared to the sham one (*p* < 0.05) likely resulted from the physiological effects of PBM. PBM helps mitochondria work better, boosting adenosine triphosphate (ATP) production and providing muscles with more energy to contract. It also improves intramuscular blood flow, raises antioxidant levels, and enhances nitric oxide release. This process improves blood and oxygen supply in tissues, along with increased neurotransmitter activity and stronger muscle fiber contraction, all of which help improve muscle function and strength (29). Additionally, stimulation of lasers to the acupoints (8), an area rich in nerve endings, triggers responses in the nervous system (30) and can increase the excitability of the central nervous system, help activate more motor units, and strengthen muscles (7), further supporting gains in trunk extensor strength.

The enhancement observed in the LILA group compared with the sham group corroborates prior research indicating that PBM enhances muscle performance and strength, particularly when applied pre-exercise (11). Specifically, at dosages of 10 and 50 J for 810 nm, there was a notable increase in voluntary quadriceps maximum contractility (31), whereas a lower dosage of 5 J was effective for the biceps brachii (32). Additionally, improvements in quadriceps and ankle dorsiflexor strength in individuals with spastic diplegia (33) and an increase in rectus femoris torque peaks in young women (34) have been reported.

The advantage of high-intensity lasers in reaching deeper tissues and generating detectable power in PBM is likely attributable to their substantial output power (≥500 mW). This power can induce additional optical vibrations within these tissues, thereby stimulating photochemical processes, such as enhanced mitochondrial activity. Consequently, this leads to increased ATP production, elevated metabolism, improved blood flow, and facilitation of edema and fluid resorption (4). These biological responses may aid in addressing capillary rarefaction, muscle perfusion deficits, and localized ischemia, conditions frequently associated with CNSLBP in deconditioned lumbar musculature (3). Together with the analgesic properties of high-intensity lasers (4), patients may experience enhanced muscle force, as evidenced by their isokinetic strength outcomes.

Previous research studies have shown the advantages of laser therapy at various intensities for improving muscle strength, particularly at high intensities. Kaydok et al. (35) demonstrated that both high- and low-intensity lasers enhanced grip strength in people with lateral epicondylitis, with a larger effect using the high-intensity laser. Several other similar studies have documented improvements in muscle strength with the use of high-intensity lasers for treating many musculoskeletal-related conditions, such as patellofemoral pain syndrome (PFPS) (36), subacromial impingement syndrome (37), and knee osteoarthritis (38). Studies specifically examining the impact of LA on trunk muscle strength in CNSLBP are limited. In addition, the minimum clinically important differences (MCID) for isokinetic parameters have not been established; therefore, clinical interpretation cannot be made and requires additional research.

Fekri et al. (39) observed no differences in gripping strength in tennis elbow when comparing high-intensity and low-intensity lasers. Various factors, including laser specifications, treatment procedures, and techniques employed, may account for these findings.

No significant variation was reported between both groups of active laser treatment, suggesting that both HILA and LILA effectively reduced the trunk extensor muscles’ fatigue. Laser therapy affects cellular metabolism, as it can penetrate the cell membrane and be absorbed by cytochrome c oxidase (mitochondrial enzymes), which stimulates ATP production and increases energy levels shortly after treatment. Additionally, the improvement of intramuscular microcirculation, reduction in lactate production, and increase in antioxidants contribute to delaying fatigue and improving muscle performance (29).

Numerous studies have demonstrated that PBM enhances oxygen delivery, prolongs the time to fatigue onset, and reduces oxidative stress and fatigue (11). It also alleviates muscle fatigue in young women (34) and decreases lactate production in the bloodstream of children with spasticity (33). Vanin et al. (40) reported that PBM, when applied prior to exercise, improved muscle performance and minimized fatigue-related signals, particularly at doses ranging from approximately 60 to 300 J for larger muscle groups with power outputs of 200 mW. The energy provided by LILA treatment was within this range.

Nonetheless, Gorgey et al. (41) failed to show the beneficial effects of low-power lasers. In their study, stimulation with neuromuscular electrical current elicited muscle tissue contractions, and laser was administered via a scanning method prior to exercise. Their scanning method might cause light to bend and energy to be lost during use, which could lower the amount of effective dose that reaches the tissue (40). In contrast, this study used lasers directly on certain acupoints, which may have made stimulation more focused and energy transfer more efficient.

While these results appear promising, understanding fatigue remains challenging due to the absence of a developed MCID for the isokinetic measurement of trunk extensor fatigue. Further research is necessary to ascertain which changes in these outcomes are clinically significant.

Both HILA and LILA led to better enhancements in pain, lumbar flexion, and disability (*p* < 0.05) compared with the sham one. The HILA group showed greater pain relief than that of the LILA group at both time points post-treatment and following up (*p* < 0.05). Pain reduction in both active laser groups had exceeded the minimum clinically important change (MCIC) of 20 mm on the VAS for LBP (26), signifying that the outcomes were not only significant statistically but also clinically important. HILA also resulted in superior lumbar flexion gains immediately post-treatment; however, these gains were not sustained at follow-up, and the clinical significance remains uncertain due to the absence of an established MCID for MST. Both HILA and LILA showed clinically meaningful improvements, surpassing the ODI MCIC’s 10 points (26). Nonetheless, no significant variation was reported among both groups at various time points, although HILA achieved a higher percentage improvement.

The reported PBM mechanisms can account for the observed enhancement in both the active laser groups. PBM can reduce pain by raising the pain threshold, blocking nociceptive transmission through A-delta and C fibers, lowering peripheral nociceptor activity, and increasing the release of natural opioids. It may also reduce inflammatory substances such as IL-1β, IL-8, TNF-α, and prostaglandins, along with lowering C-reactive protein levels. This contributes to pain relief and reduced inflammation, improves the underlying disease, and may help with functional recovery (13). PBM may further reduce inflammation and improve muscle function when combined with exercise, resulting in decreased pain and impairment (42). Previous studies have shown that an 808 nm infrared GaAlAs laser reduced pain and disability in CNSLBP (13). Pain improvement has also been observed in children with spastic diplegia (33).

Another reason for the observed benefits was the LA. Stimulating specific acupoints with laser therapy (8) may activate more mechanisms for pain relief by controlling the gate theory and causing the body to release natural opioids. BL23, BL24, and BL26 may help reduce myofascial problems and improve local blood flow. Simultaneously, BL25 and BL40 effectively increased local blood flow and reduced muscle tightness. The GV3 point may influence muscle activity, helping to alleviate issues such as lumbar weakness and sciatic pain (30). These acupoints were used in the current study. Previous research has shown that acupuncture can reduce pain, improve spinal ROM, and decrease dysfunction in CNSLBP (43). It also reduces pain and functional impairment (6), especially when combined with LA (8, 16). Allam et al. (44) found that LA improved pain, knee ROM, and function in PFPS. Additionally, Mao et al. (45) found that LA provides immediate pain relief for chronic LBP, although this relief may be temporary. Thus, the use of LA may have improved the benefits of both laser techniques.

High-intensity laser acupuncture provides superior pain relief for several reasons. High-intensity lasers may increase beta-endorphins and other bodily painkillers in the CNS, which lessens pain. At the tissue level, it might lessen hyperalgesia, decrease substance P release, reduce nerve conduction velocity, and lengthen reaction times to reduce pain transmission signals. It may also lower inflammatory mediators, such as bradykinin and histamine, which increases pain tolerance. When high energy is delivered to tissues, it creates kinetic optical vibrations that cause photochemical reactions, speed up circulation and metabolism, and facilitate the absorption of congestion and interstitial fluid. Additionally, the short, rapid pulses of this laser produce pressure waves inside the tissues that suppress nociceptive nerve terminals and activate free nerve endings, both of which help reduce pain. High-intensity lasers also cause mild surface warming and vasodilation, enhance local blood flow, and encourage muscle release (4). This effect could account for the greater increase in the lumbar flexion range in the HILA group. However, this benefit might only last temporarily, as this advantage was not sustained at follow-up.

High-intensity lasers were reported to be better than low-intensity lasers in reducing pain associated with knee osteoarthritis (46) and plantar fasciitis (47). These lasers have also been shown to improve pain in various conditions, including PFPS (36), knee osteoarthritis (38), postoperative incisional pain (48), and subacromial impingement syndrome, where they enhance pain relief, ROM, and functional outcomes, both immediately and over time (37). Furthermore, high-intensity light is considered a noninvasive treatment for chronic LBP, offering benefits for pain relief, lumbar ROM, and functional ability, particularly when combined with exercise (4). Although a recent review found no significant variation between high- and low-intensity lasers in alleviating pain for several musculoskeletal disorders, including LBP, high-intensity lasers provide additional skeletal benefits, such as increased muscle thickness and improved echogenicity (9). Similarly, Chen et al. (10) observed that both laser strategies improved pain and functional outcomes in the short term in individuals with persistent LBP.

Patients with lateral epicondylitis experienced a decrease in pain following treatment with both high- and low-intensity lasers; however, the improvement percentage was larger in the high-intensity group (35). Variations in treatment time, light dosage, and application parameters may be the cause of the differences observed in comparison to the present research. Similarly, Abdelbasset et al. (13) reported no appreciable differences when comparing the two lasers in terms of lumbar ROM and discomfort in CNSLBP. This variation could be explained by the light used in their experiment, which included varying treatment doses, applications, and time intervals.

Although the HILA group demonstrated a higher percentage of improvement, both laser intensities provided similar improvements in impairment, with no apparent distinction between the HILA and LILA groups at post-treatment or follow-up. This result agrees with the results of Abdelbasset et al. (13), who found advantages with both modalities but no discernible superiority. Ezzati et al. (49) reported that after 1 month, both laser intensities enhanced the ability to function in knee arthritis, with the high-intensity group showing the greatest improvement. In contrast, in cases of lateral epicondylitis (35), plantar fasciitis (47), and knee osteoarthritis (46), high-intensity lasers produced better functional results than low-intensity lasers. The underlying clinical problems and variances in patient characteristics could be the cause of these discrepancies.

However, in short- and long-term follow-ups, Taradaj et al. (50) found that either high- or low-intensity lasers were ineffective in treating degenerative changes of the lumbar disk and did not significantly improve lumbar mobility, reduce discomfort, or improve functional status when compared to a placebo. This disparity may be due to differences in treatment regimens, light dosage, and patient circumstances. Furthermore, Glazov et al. (51) observed no notable variation in pain and disability between the LA or sham groups and that all groups benefited independently of laser stimulation. The significantly low parameters of a laser used in their investigation, which applied small doses (0.2–0.8 J per point) as well as low power output (20 mW), contrast with the greater energy used in either high- or low-intensity lasers that were used in the present research, which might have generated stronger PBM effects. Similarly, Shin et al. (16) found no variation between LA and sham therapy for LBP; nevertheless, their therapy duration was only a week, in contrast to the current study’s 4-weeks treatment period, which may have allowed LA’s analgesic effects to develop.

The positive results for the sham group probably stemmed from the placebo effect and the supervised exercise program that everyone followed. According to Qaseem et al. (5), the ACP frequently recommends exercise therapy for persistent LBP, as it improves function and lessens discomfort. It has been demonstrated that lumbar stability and kinetic strengthening exercises increase the trunk strength levels and reduce impairment and pain in those with chronic lower back pain (21). Additionally, core stability exercises improve trunk muscle activation and motor control, which helps reduce discomfort and impaired function (52). According to previous research, core stability training regimens can greatly reduce LBP, exhaustion, back muscular endurance, and impairment (53). In addition, stretching exercises increase stretch tolerance and decrease muscular stiffness, which enhances ROM (20).

## Strengths and limitations

There were various strengths of the present study. Its sham-controlled, randomized, and double-blind design minimizes possible bias and strengthens the internal validity of the results of the study. Furthermore, it evaluates the relative efficacy of different laser intensities in treating CNSLBP by directly comparing high- and low-intensity LA with supervised exercise therapy. The inclusion of a sham group reduced the placebo effect, allowing a clearer interpretation of the treatment outcomes. A key advantage of this research is the objective evaluation of trunk muscular strength and fatigue through an isokinetic-based assessment, which provides a more comprehensive analysis than previous studies that primarily focused on pain, lumbar ROM, and impairment outcomes. Additionally, the rapid application of high-intensity lasers may offer practical benefits in therapeutic settings.

However, there are certain limitations that should be acknowledged. The short follow-up period limits the conclusions regarding the long impact of these effects. Although all participants were analyzed and completed the study, the evaluation was not predetermined as an intention-to-treat analysis. The narrow age and BMI ranges may restrict the generalizability of the results. As all groups had received the same exercise program, the independent effect of exercise treatment could not be distinguished from that of LA treatment. Psychosocial variables like avoidance of fear beliefs, depression, anxiety, and pain catastrophizing were not assessed despite their potential influence on pain, disability, and treatment responses. The use of medications during follow-up was not systematically documented. Although quantitative isokinetic assessment was employed, other objective measurements, such as electromyography or ultrasound imaging, which may have provided more about neuromuscular response, were not performed. Although high-intensity lasers demonstrate superior clinical effects, their high cost and the absence of standard therapy protocols may hinder their application in many clinical settings. Future studies should incorporate longer follow-up periods, intention-to-treat analyses, broader populations, assessment of psychosocial factors and medication use, and assessment of optimal laser parameters and their cost-effectiveness.

## Conclusion

In patients with CNSLBP, both HILA and LILA, when combined with exercise, appear to be safe and noninvasive interventions. These methods provide superior short-term improvements in trunk extensor strength and fatigue, pain, lumbar flexion ROM, and disability compared with exercise alone. HILA may be more effective than LILA in enhancing trunk strength and alleviating pain. These findings suggest that incorporating LA, particularly HILA, into exercise regimens may yield additional clinical benefits. Further research is necessary to determine the optimal parameters and long-term effects.

## Data Availability

The raw data supporting the conclusions of this article will be made available by the authors, without undue reservation.

## Footnotes

1
www.randomization.com

## References

[1] Reyes-Ferrada W Chirosa-Rios L Rodriguez-Perea A Jerez-Mayorga D Chirosa-Rios I. Isokinetic trunk strength in acute low back pain patients compared to healthy subjects: a systematic review. *Int J Environ Res Public Health.* (2021) 18:2576. 10.3390/ijerph18052576 33806622 PMC7967351

[2] Hodges P Tucker K. Moving differently in pain: a new theory to explain the adaptation to pain. *Pain.* (2011) 152:S90–8. 10.1016/j.pain.2010.10.020 21087823

[3] Valdivieso P Franchi M Gerber C Flück M. Does a better perfusion of deconditioned muscle tissue release chronic low back pain? *Front Med.* (2018) 5:77. 10.3389/fmed.2018.00077 29616222 PMC5869187

[4] Pramesti A Adhitya A. The effect of high intensity laser therapy on low back pain: a literature review. *Int J Med Sci Clin Res Stud.* (2025) 5:1713–8. 10.47191/ijmscrs/v5-i10-11

[5] Qaseem A Wilt T McLean R Forciea M Clinical Guidelines Committee of the American College of Physicians. Noninvasive treatments for acute, subacute, and chronic low back pain: a clinical practice guideline from the American college of physicians. *Ann Intern Med.* (2017) 166:514–30. 10.7326/M16-2367 28192789

[6] Asano H Plonka D Weeger J. Effectiveness of acupuncture for nonspecific chronic low back pain: a systematic review and meta-analysis. *Med Acupunct.* (2022) 34:96–106. 10.1089/acu.2021.0057 35509875 PMC9057891

[7] Wang I Chen Y Hu R Wang J Li Z. Effect of acupuncture on muscle endurance in the female shoulder joint: a pilot study. *Evid Based Complement Alternat Med.* (2020) 2020:9786367. 10.1155/2020/9786367 32952592 PMC7487087

[8] Wu D Zhao Y Dai R Rong P Wang Y. Application of photobiomodulation therapy in acupuncture. *World J Tradit Chin Med.* (2022) 8:491. 10.4103/wjtcm.wjtcm_12_22 23885341 PMC3676159

[9] Saleh M Shahien M Mortada H Elaraby A Hammad Y Hamed M et al. High-intensity versus low-level laser in musculoskeletal disorders. *Lasers Med Sci.* (2024) 39:179. 10.1007/s10103-024-04111-1 38990213 PMC11239763

[10] Chen Y Liao C Hong J Hsu W Wu C Chen H. Effects of laser therapy on chronic low back pain: a systematic review and meta-analysis of randomized controlled trials. *Clin Rehabil.* (2022) 36:289–302. 10.1177/02692155211057435 34757882

[11] Oliveira A Silva JL Camillo CAM Andraus RAC Maia LP. Does photobiomodulation improve muscle performance and recovery? a systematic review. *Rev Bras Med Esporte.* (2022) 29:1–7. 10.1590/1517-8692202329012021_0412

[12] Liu R Moe A Zheng Z Alanazi H Zoghi M Jaberzadeh S. Effects of laser acupuncture on pain and motor function in musculoskeletal disorders: a systematic review and meta-analysis. *Complement Ther Med.* (2026) 96:103323. 10.1016/j.ctim.2026.103323 41547416

[13] Abdelbasset W Nambi G Alsubaie S Abodonya A Saleh A Ataalla N et al. randomized comparative study between high-intensity and low-level laser therapy in the treatment of chronic nonspecific low back pain. *Evid Based Complement Alternat Med.* (2020) 2020:1350281. 10.1155/2020/1350281 33178306 PMC7644303

[14] Custers P Van de Kelft E Eeckhaut B Sabbe W Hofman A Debuysscher A et al. Clinical examination, diagnosis, and conservative treatment of chronic low back pain: a narrative review. *Life.* (2024) 14:1090. 10.3390/life14091090 39337874 PMC11433180

[15] Shokri P Zahmatyar M Falah Tafti M Fathy M Rezaei Tolzali M Ghaffari Jolfayi A et al. Non-spinal low back pain: global epidemiology, trends, and risk factors. *Health Sci Rep.* (2023) 6:e1533. 10.1002/hsr2.1533 37674621 PMC10477419

[16] Shin J Ku B Kim J Lee Y Kang J Heo H et al. Short-term effect of laser acupuncture on lower back pain: a randomized, placebo-controlled, double-blind trial. *Evid Based Complement Alternat Med.* (2015) 2015:808425. 10.1155/2015/808425 26516333 PMC4606147

[17] Kim G Kim D Moon H Yoon D Lee S Ko S et al. Acupuncture and acupoints for low back pain: systematic review and meta-analysis. *Am J Chin Med.* (2023) 51:223–47. 10.1142/S0192415X23500131 36585839

[18] World Health Organization. *World Health Organization Regional Office for the Western Pacific. WHO Standard Acupuncture Point Locations in the Western Pacific Region.* Geneva: World Health Organization (2008).

[19] Elnaggar I Nordin M Sheikhzadeh A Parnianpour M Kahanovitz N. Effects of spinal flexion and extension exercises on low-back pain and spinal mobility in chronic mechanical low-back pain patients. *Spine.* (1991) 16:967–72. 10.1097/00007632-199108000-00018 1835157

[20] Page P. Current concepts in muscle stretching for exercise and rehabilitation. *Int J Sports Phys Ther.* (2012) 7:109–19. 22319684 PMC3273886

[21] Moon H Choi K Kim D Kim H Cho Y Lee K et al. Effect of lumbar stabilization and dynamic lumbar strengthening exercises in patients with chronic low back pain. *Ann Rehabil Med.* (2013) 37:110. 10.5535/arm.2013.37.1.110 23525973 PMC3604220

[22] Gabr W Eweda R. Isokinetic strength of trunk flexors and extensors muscles in adult men with and without nonspecific back pain: a comparative study. *J Behav Brain Sci.* (2019) 9:340–50. 10.4236/jbbs.2019.99025

[23] García-Vaquero M Barbado D Juan-Recio C López-Valenciano A Vera-Garcia F. Isokinetic trunk flexion-extension protocol to assess trunk muscle strength and endurance: reliability, learning effect, and sex differences. *J Sport Health Sci.* (2020) 9:692–701. 10.1016/j.jshs.2016.08.011 33308821 PMC7749212

[24] Brown L. *Isokinetics in Human Performance.* Champaign, IL: Human Kinetics (2000).

[25] Ghasemi C Amiri A Sarrafzadeh J Jafari H Dadgoo M. The effect of soft tissue manipulation and rest on knee extensor muscles fatigue: do torque parameters and induced perception following muscle fatigue have enough reliability? *J Fam Med Prim Care.* (2020) 9:950–6. 10.4103/jfmpc.jfmpc_838_19 32318451 PMC7114034

[26] Ostelo R de Vet H. Clinically important outcomes in low back pain. *Best Pract Res Clin Rheumatol.* (2005) 19:593–607. 10.1016/j.berh.2005.03.003 15949778

[27] Rezvani A Ergin O Karacan I Oncu M. Validity and reliability of the metric measurements in the assessment of lumbar spine motion in patients with ankylosing spondylitis. *Spine.* (2012) 37:E1189–96. 10.1097/BRS.0b013e31825ef954 22614802

[28] Algarni A Ghorbel S Jones J Guermazi M. Validation of an Arabic version of the Oswestry index in Saudi Arabia. *Ann Phys Rehabil Med.* (2014) 57:653–63. 10.1016/j.rehab.2014.06.006 25262247

[29] Cubas I Eckert J Canalli L Carvalho A Bertolini G. Photobiomodulation in aspects of muscle function: a scoping review. *J Pre Clin Clin Res.* (2023) 17:32–6. 10.26444/jpccr/161689

[30] Lim T Ma Y Berger F Litscher G. Acupuncture and neural mechanism in the management of low back pain—an update. *Medicines.* (2018) 5:63. 10.3390/medicines5030063 29941854 PMC6164863

[31] Vanin A De Marchi T Tomazoni S Tairova O Leão Casalechi H de Tarso Camillo de Carvalho P et al. Pre-exercise infrared low-level laser therapy (810 nm) in skeletal muscle performance and postexercise recovery in humans, what is the optimal dose? a randomized, double-blind, placebo-controlled clinical trial. *Photomed Laser Surg.* (2016) 34:473–82. 10.1089/pho.2015.3992 27575834

[32] Almeida P Lopes-Martins RÁB De Marchi T Tomazoni SS Albertini R Corrêa JC et al. Red (660 nm) and infrared (830 nm) low-level laser therapy in skeletal muscle fatigue in humans: what is better? *Lasers Med Sci.* (2012) 27:453–8. 10.1007/s10103-011-0957-3 21814736 PMC3282894

[33] Abdelhalim S Shoukry K Alsharnoubi J. Effect of low-level laser therapy on quadriceps and foot muscle fatigue in children with spastic diplegia: a randomized controlled study. *Lasers Med Sci.* (2023) 38:182. 10.1007/s10103-023-03841-y 37572215 PMC10423123

[34] Toma R Oliveira M Renno A Laakso E. Photobiomodulation (PBM) therapy at 904 nm mitigates effects of exercise-induced skeletal muscle fatigue in young women. *Lasers Med Sci.* (2018) 33:1197–205. 10.1007/s10103-018-2454-4 29455305

[35] Kaydok E Ordahan B Solum S Karahan A. Short-term efficacy comparison of high-intensity and low-intensity laser therapy in the treatment of lateral epicondylitis: a randomized double-blind clinical study. *Arch Rheumatol.* (2019) 35:60–7. 10.5606/ArchRheumatol.2020.7347 32637921 PMC7322301

[36] Ozlu O Atilgan E. The effect of high-intensity laser therapy on pain and lower extremity function in patellofemoral pain syndrome: a single-blind randomized controlled trial. *Lasers Med Sci.* (2024) 39:103. 10.1007/s10103-024-04017-y 38630331 PMC11024020

[37] Yılmaz M Eroglu S Dundar U Toktas H. The effectiveness of high-intensity laser therapy on pain, range of motion, functional capacity, quality of life, and muscle strength in subacromial impingement syndrome: a 3-month follow-up, double-blinded, randomized, placebo-controlled trial. *Lasers Med Sci.* (2022) 37:241–50. 10.1007/s10103-020-03224-7 33400012

[38] Tangsriwong K Sakulsriprasert P Bunprajun T Thammajaree C Ariyakitsakul N. Effects of single-session high-intensity laser therapy on knee pain, joint position sense, and muscle strength in individuals with knee osteoarthritis: a pilot randomized controlled trial. *J Musculoskelet Surg Res.* (2025) 9:354–60. 10.25259/jmsr_32_2025

[39] Fekri L Rezvani A Karimi N Ezzati K. The effect of low-power and high-power laser therapy on pain, tenderness, and grip force of the patients with tennis elbow. *Pharmacophore.* (2019) 10:89–95.

[40] Vanin A Verhagen E Barboza S Costa L Leal-Junior E. Photobiomodulation therapy for the improvement of muscular performance and reduction of muscular fatigue associated with exercise in healthy people: a systematic review and meta-analysis. *Lasers Med Sci.* (2018) 33:181–214. 10.1007/s10103-017-2368-6 29090398

[41] Gorgey A Wadee A Sobhi N. The effect of low-level laser therapy on electrically induced muscle fatigue: a pilot study. *Photomed Laser Surg.* (2008) 26:501–6. 10.1089/pho.2007.2161 18922091

[42] Oliveira M Bjordal JM Schardong J Plentz RDM Casalechi HL Leal-Junior ECP et al. Effects of photobiomodulation therapy associated with motor control exercise for chronic non-specific low back pain: protocol for a randomised placebo-controlled trial. *BMJ Open Sport Exerc Med.* (2024) 10:e002199. 10.1136/bmjsem-2024-002199 39345834 PMC11429343

[43] Xu F Jiao K Wang L Zhang F. Acupuncture for chronic nonspecific low back pain in middle-aged and older patients: a randomized controlled trial. *World J Acupunct Moxibustion.* (2024) 34:300–5. 10.1016/j.wjam.2024.09.007

[44] Allam N Alsirhani H Alruwaili M Dosh D Alruwaili H Almazyad W et al. Effect of laser acupuncture on pain, range of motion, and function in patellofemoral pain syndrome: a randomised controlled trial. *Front Med.* (2025) 12:1613197. 10.3389/fmed.2025.1613197 40735438 PMC12303989

[45] Mao X He H Ding J. Efficacy of laser acupuncture for treatment of chronic low back pain: a systematic review and meta-analysis. *Pain Manag Nurs.* (2024) 25:529–37. 10.1016/j.pmn.2024.05.001 38821755

[46] Kheshie A Alayat M Ali M. High-intensity versus low-level laser therapy in the treatment of patients with knee osteoarthritis: a randomized controlled trial. *Lasers Med Sci.* (2014) 29:1371–6. 10.1007/s10103-014-1529-0 24487957

[47] Ordahan B Karahan A Kaydok E. The effect of high-intensity versus low-level laser therapy in the management of plantar fasciitis: a randomized clinical trial. *Lasers Med Sci.* (2018) 33:1363–9. 10.1007/s10103-018-2497-6 29627888

[48] Ibrahim A Awad M El Refaye G Gabr A Mahmoud M. Effect of high level laser therapy on postoperative cesarean section incisional pain. *Benha Int J Phys Ther.* (2025):121–30. 10.21608/bijpt.2025.403294.1104

[49] Ezzati K Esmaili K Reihanian Z Hasannejad A Soleymanha M Keshavarz S et al. The effects of high-intensity laser therapy vs. low-level laser therapy on functional ability and quadriceps architecture in patients with knee osteoarthritis: a single-blinded randomized clinical trial. *J Lasers Med Sci.* (2024) 15:e66. 10.34172/jlms.2024.66 39949479 PMC11822233

[50] Taradaj J Rajfur K Shay B Rajfur J Ptaszkowski K Walewicz K et al. Photobiomodulation using high- or low-level laser irradiations in patients with lumbar disc degenerative changes: disappointing outcomes and remarks. *Clin Interv Aging.* (2018) 13:1445–55. 10.2147/CIA.S168094 30174418 PMC6109659

[51] Glazov G Yelland M Emery J. Low-dose laser acupuncture for non-specific chronic low back pain: a double-blind randomised controlled trial. *Acupunct Med.* (2014) 32:116–23. 10.1136/acupmed-2013-010456 24280948 PMC3995277

[52] Puntumetakul R Areeudomwong P Emasithi A Yamauchi J. Effect of 10-week core stabilization exercise training and detraining on pain-related outcomes in patients with clinical lumbar instability. *Patient Prefer Adherence.* (2013) 7:1189–99. 10.2147/PPA.S50436 24399870 PMC3875242

[53] Baza M Amin F Ibrahim A. Effect of biomechanical awareness and core stability exercises on mechanical low back pain among Egyptian physiotherapists. *Benha Int J Phys Ther.* (2024) 2:75–86. 10.21608/BIJPT.2024.336636.1046

