# Supplementary material for: High-intensity versus low-intensity laser acupuncture in chronic, non-specific low back pain: a double-blinded, randomized controlled trial
Source: Front Med (Lausanne). 2026 Jul 15;13:1857622. doi: 10.3389/fmed.2026.1857622 (PMC13416075; doi:10.3389/fmed.2026.1857622)
Supplement: Supplementary file 1 [file Table_1.docx]

**Supplementary TABLE 1.** Bonferroni-adjusted pairwise comparisons of peak torque, fatigue, pain, lumbar flexion ROM, and disability at the pre, post, and follow-up assessments among the three groups.

| **Variables** | **HILA group (n=20)** | | | **LILA group (n=20)** | | | **Sham group (n=20)** | | |
| --- | --- | --- | --- | --- | --- | --- | --- | --- | --- |
|  | **MD (95%CI)** | **% of change** | ***p* -value** | **MD (95%CI)** | **% of change** | ***p* -value** | **MD (95%CI)** | **% of change** | ***p* -value** |
| **Peak torque (N.m)** | | | | | | | | | |
| **Pre Vs. Post** | 36.95  (31.95,41.96) | 31.38% | <0.001^*^ | 23.36 (19.81,26.90) | 19.51% | <0.001^*^ | 12.75 (9.98,15.53) | 10.72% | <0.001^*^ |
| **Pre Vs. Follow-up** | 35.25 (30.30,40.20) | 29.94% | <0.001^*^ | 22.22 (18.71,25.72) | 18.56% | <0.001^*^ | 11.20 (8.28,14.12) | 9.41% | <0.001^*^ |
| **Post Vs. Follow-up** | -1.71  (-3.17, -0.24) | 1.09% | 0.007^*^ | -1.14  (-1.56, -0.72) | 0.79% | <0.001^*^ | -1.55  (-2.08, -1.03) | 1.18% | <0.001^*^ |
| **Fatigue (Reps)** | | | | | | | | | |
| **Pre Vs. Post** | 18.70  (14.73,22.67) | 69.52% | <0.001^*^ | 15.40 (11.44,19.36) | 54.22% | <0.001^*^ | 9.20 (6.71,11.69) | 33.45% | <0.001^*^ |
| **Pre Vs. Follow-up** | 16.70 (12.69,20.71) | 62.08% | <0.001^*^ | 13.50 (9.39,17.61) | 47.54% | <0.001^*^ | 7.35 (5.09,9.61) | 26.72% | <0.001^*^ |
| **Post Vs. Follow-up** | -2.00  (-2.54, -1.46) | 4.39% | <0.001^*^ | −1.90  (-2.56, -1.24) | 4.34% | <0.001* | -1.85  (-2.59,-1.11) | 5.04% | <0.001* |
| **Pain (mm)** | | | | | | | | | |
| **Pre Vs. Post** | −41.15  (-47.20,-35.10) | 59.25% | <0.001^*^ | −35.55  (-42.94,-28.16) | 50.21% | <0.001* | -13.15  (-16.12,-10.18) | 19.32% | <0.001* |
| **Pre Vs. Follow-up** | −34.75  (-41.64,-27.86) | 50.04% | <0.001^*^ | −29.85  (-36.60,-23.10) | 42.16% | <0.001^*^ | -8.20  (-11.09,-5.31) | 12.05% | <0.001^*^ |
| **Post Vs. Follow-up** | 6.40  (2.81,9.99) | 22.61% | <0.001^*^ | 5.70 (2.68,8.72) | 16.17% | <0.001^*^ | 4.95 (2.40,7.50) | 9.02% | <0.001^*^ |
| **ROM Flex (cm)** | | | | | | | | | |
| **Pre Vs. Post** | 2.39  (2.09,2.69) | 59.31% | <0.001^*^ | 1.69 (1.30,2.09) | 39.21% | <0.001^*^ | 1.12 (0.82,1.42) | 26.29% | <0.001^*^ |
| **Pre Vs. Follow-up** | 2.10  (1.84,2.36) | 52.11% | <0.001^*^ | 1.53 (1.11,1.95) | 35.49% | <0.001^*^ | 0.86 (0.56,1.16) | 20.19% | <0.001^*^ |
| **Post Vs. Follow-up** | -0.29  (-0.38,-0.21) | 4.517% | <0.001^*^ | -0.17  (-0.31,-0.01) | 2.67% | 0.024^*^ | -0.26  (-0.36,-0.16) | 4.83% | <0.001^*^ |
| **Disability (%)** | | | | | | | | | |
| **Pre Vs. Post** | -14.70  (-17.61,-11.79) | 42.79% | <0.001^*^ | -10.50  (-13.24, -7.76) | 32.26% | <0.001^*^ | -5.05  (-6.72,-3.38) | 15.14% | <0.001^*^ |
| **Pre Vs. Follow-up** | -13.45  (-16.55,-10.35) | 39.16% | <0.001^*^ | -8.55  (-11.45,-5.65) | 26.27% | <0.001^*^ | -3.30  (-5.09,-1.51) | 9.89% | <0.001^*^ |
| **Post Vs. Follow-up** | 1.25  (0.88, 1.62) | 6.361% | <0.001^*^ | 1.95 (1.20,2.70) | 8.84% | <0.001^*^ | 1.75 (0.88,2.62) | 6.18% | <0.001^*^ |

MD, mean difference; CI, confidence interval; *, Statistically significant at *p* -value < 0.05.
